# A rare association of pulmonary aplasia with cervicomedullary junction anomalies: a clinical image

**DOI:** 10.11604/pamj.2025.52.95.48957

**Published:** 2025-11-04

**Authors:** Vaishnavi Yadav, Pallavi Harjpal

**Affiliations:** 1 Department of Cardiovascular and Respiratory Physiotherapy, Ravi Nair Physiotherapy College, Datta Meghe Institute of Higher Education and Research, Sawangi (Meghe), Wardha, Maharashtra, India,; 2 Department of Neurophysiotherapy, Ravi Nair Physiotherapy College, Datta Meghe Institute of Higher Education and Research, Sawangi (Meghe), Wardha, Maharashtra, India

**Keywords:** Pulmonary hypoplasia, complete lung atelectasis, pulmonary agenesis

## Image in medicine

Pulmonary aplasia is a rare congenital anomaly characterized by the complete absence of the lung parenchyma and bronchial structures, resulting from failure of the lung bud to develop. Its estimated incidence is 1-3.4 per 100,000 live births, and it is often associated with malformations involving the cardiovascular, gastrointestinal, genitourinary, or musculoskeletal systems. We present the case of a 20-year-old female who reported a one-month history of tingling and numbness in both upper limbs, significantly limiting daily activities. She also experienced a cough with expectoration and progressive shortness of breath. Cervical spine magnetic resonance imaging (MRI) demonstrated partial block vertebrae at C2-C3 and C4-C5, kinking at the cervicomedullary junction with cord thinning, subtle intramedullary edema, and the presence of a syrinx (Panel A, yellow arrow). Chest radiography revealed a dense opacity occupying the right hemithorax, accompanied by ipsilateral tracheal shift, hyperinflation of the contralateral lung, widening of intercostal spaces, and thoracic scoliosis (Panel B, green arrow). In the MRI, narrowing of the foramen magnum further indicated a significant craniocervical junction anomaly (Panel C, red arrow). This combination of abnormalities represents a multisystem developmental disorder, likely stemming from an early embryological insult involving both foregut derivatives and the paraxial mesoderm. The case highlights the need for a comprehensive, systematic evaluation for associated anomalies in patients with pulmonary aplasia, even when the primary presentation is neurological.

**Figure 1:**
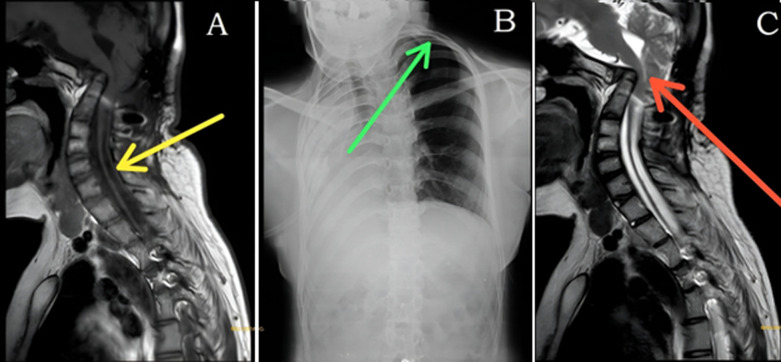
A) yellow arrow indicates subtle edema and syrinx formation; B) green arrow indicates lung hyperinflation and intercostal widening; C) red arrow indicates cervicomedullary kinking and thinning; narrowing of the foramen magnum was noted

